# Exploring Fat Fraction and Vertebral Bone Quality Score in Lumbar Spine Magnetic Resonance Imaging: A Cross-Sectional Study on Associations and Clinical Implication

**DOI:** 10.3390/diagnostics15040503

**Published:** 2025-02-19

**Authors:** Sunghoon Park, Jinwoo Hwang, Kyu-Sung Kwack, Kyu Hong Lee, Jae Sung Yun

**Affiliations:** 1 Department of Radiology, Ajou University School of Medicine, Suwon 16499, Republic of Korea; 2 Musculoskeletal Imaging Laboratory, Ajou University Medical Center, Suwon 16499, Republic of Korea; 3 Philips Healthcare, Seoul 04637, Republic of Korea; 4 Department of Radiology, College of Medicine, Inha University, Incheon 22332, Republic of Korea

**Keywords:** MRI, fat fraction, vertebral bone quality score, spine

## Abstract

**Background/Objectives:** While gradient-echo (GRE)-based chemical shift-encoded magnetic resonance imaging (CSE-MRI) offers precise method for measuring adiposity in bone marrow, its limitation lies in the need for additional imaging. On the other hand, spin-echo (SE)-based CSE-MRI can seamlessly integrate into conventional protocols. Recently, a novel technique called the vertebral bone quality (VBQ) score has been introduced. The objective of this study was to investigate the association between fat fraction (FF) measured by GRE-based CSE-MRI (FF_GRE_) and FF measured by SE-based CSE-MRI (FF_SE_) or the VBQ score. **Methods:** A retrospective study with 344 patients assessed the correlation between FF and the VBQ score and each measurement’s correlation with age using Pearson’s correlation (*r*). Concordance between FF_GRE_ and FF_SE_ was assessed using Lin’s concordance correlation coefficient (ρ_c_). Vertebral lesions (*n* = 41) were categorized as benign and malignant, and the Mann–Whitney U test was used for comparison. **Results:** FF_GRE_ demonstrated strong positive correlations with FF_SE_ and the VBQ score (*r* = 0.861 and 0.708, respectively). However, the concordance between FF_GRE_ and FF_SE_ was poor (ρ_c_ = 0.295). All measurements moderately correlated with age (FF_GRE_, *r* = 0.583; FF_SE_, *r* = 0.477; VBQ score, *r* = 0.468). FF was significantly higher in benign lesions (FF_GRE_, *p* = 0.004; FF_SE_, *p* = 0.007), while the VBQ score did not show statistically significant differences between the two groups (*p* = 0.089). **Conclusions:** FF_GRE_ exhibited a high correlation with the VBQ score. FF_SE_ showed a strong correlation with FF_GRE_, but replacing FF_GRE_ with FF_SE_ may be challenging. Both FF and the VBQ score moderately correlated with age. FF demonstrated statistically significant differences between benign and malignant lesions, while the VBQ score did not provide a distinguishable separation.

## 1. Introduction

Bone marrow adipose tissue (BMAT) is a crucial organ that influences not only hematopoiesis but also plays a significant role in the areas of immune function, paracrine signaling, and endocrine regulation [1,2,3]. Adipogenesis in the bone marrow can undergo changes based in the body’s conditions, such as aging, menopause, diabetes mellitus, anorexia nervosa, hematopoietic disease, osteoporosis, and other health-related factors [1,2,3,4,5]. Therefore, assessing the quantity of BMAT can serve as a clue to estimate the body’s condition.

Chemical shift-encoded magnetic resonance imaging (CSE-MRI) is a technique that allows for the generation of four different images (in-phase, out-of-phase, water-only, and fat-only) in a single acquisition. In addition, gradient-echo (GRE)-based CSE-MRI can easily measure the quantity of BMAT by creating a fat fraction (FF) map [6]. It is well known that there are many confounding factors when calculating the signal of water or fat using CSE-MRI. For example, trabecular bone shortens the T2* of water and fat components, making it difficult to separate water and fat signals. Therefore, correcting for T2* decay is crucial when quantifying fat in bone marrow [7]. The smaller signals from peaks other than the largest peak at −3.5 ppm in the fat spectrum are easily overlooked [7]. The T1 bias resulting from the difference in T1 relaxation times between water and fat also acts as a confounding factor [8]. The FF measured using multi-echo GRE-based CSE-MRI, by correcting for these confounding factors, has been reported to closely match results obtained from magnetic resonance spectroscopy, which is known for its high accuracy [9,10]. There have been studies aiming to apply FF measurements in various fields, such as estimating osteoporosis [11,12] or distinguishing between benign and malignant lesions [13,14]. However, GRE-based CSE-MRI comes with the inconvenience of requiring additional imaging beyond conventional MRI.

Spin-echo (SE)-based CSE-MRI can be utilized with conventional sequences due to its higher signal-to-noise ratio [15]. Furthermore, SE-based CSE-MRI has the advantage of achieving homogeneous fat suppression and reducing imaging time. This technique also generates the same four image sets as GRE-based CSE-MRI (in-phase, out-of-phase, water-only and fat-only), and, although it does not fully correct for confounding factors, it theoretically allows for the creation of a fat fraction map for measuring BMAT. Therefore, despite various limitations, we hypothesized that the fat fraction measured by SE-based CSE-MRI could potentially replace GRE-based CSE MRI.

Recently, a method called the vertebral bone quality (VBQ) score has been devised for assessing bone density using MRI [16,17,18,19,20,21,22]. This method estimates bone density through the utilization of an increase in T1 signal intensity associated with the escalation of BMAT in osteoporosis, using MRI, and shares similarities in principle with FF assessment. However, to the best of our knowledge, the relationship between FF and the VBQ score has not been directly compared. Additionally, despite using the same principle, the VBQ score has not been utilized for differentiating benign and malignant lesions. If the VBQ score has the ability to differentiate between the two lesions, it could serve as an additional tool for distinguishing indeterminate lesions and providing diagnostic confidence when conventional imaging alone is insufficient. Therefore, in our study, we aimed to use GRE-based CSE-MRI as the gold standard to investigate the relationship between SE-based CSE-MRI or the VBQ score. We investigated whether there is a correlation with aging for the three techniques and explored their ability to distinguish between benign and malignant lesions.

## 2. Materials and Methods

### 2.1. Patient Selection

This retrospective study was approved by our institutional review board, and the requirement for informed consent was waived. Between January 2021 and February 2022, patients who underwent lumbar spine MRI were included in the study. The inclusion criteria were patients who underwent MRI that included both GRE- and SE-based CSE-MRI as well as T1-weighted images. The exclusion criteria were as follows: (1) errors in GRE- or SE-based CSE-MRI; (2) history of hematopoietic diseases; (3) structural abnormalities in two or more vertebra due to fracture, severe degeneration, infection, tumor or previous surgery; (4) previous radiation treatment.

### 2.2. Magnetic Resonance Imaging

The lumbar spine MRI was conducted using a 3-T MRI scanner (Ingenia Elition X, Philips Healthcare, Best, The Netherlands). Initially, conventional MRI was obtained, including sagittal T1-weighted SE (repetition time [TR]/echo time [TE], 400–650 ms/10–15 ms) and flexible 2-point sagittal mDixon-XD T2-weighted SE (TR/echo spacing/delta TE, 2000–2900 ms/90–100 ms/6 ms). The other parameters of sagittal images were as follows: field of view (FOV), 280 × 280 mm; matrix, 280 × 240–510 × 310; section thickness, 3.5 mm; intersection gap, 0.5–0.35 mm. Axial T1-weighted SE (TR/TE, 500–720 ms/10 ms) and T2-weighted SE (TR/TE, 3300–5600 ms/80–120 ms) were also included, and the parameters were as follows: FOV, 240 × 240 mm; matrix, 340–370 × 330–370; section thickness, 4 mm; intersection gap, 0.4 mm.

Subsequently, 3D GRE-modified CSE-MRI (mDixon Quant, Philips Healthcare, Best, The Netherlands) were obtained. The imaging parameters were as follows: TR, 8 ms; six TEs (echo spacing/delta TE, 1.26 ms/1.0 ms); flip angle, 3°, FOV, 280 × 280 mm; matrix, 192 × 192; section thickness, 3.85 mm; intersection gap, 0 mm. Following acquisition, each image was reconstructed automatically and simultaneously into the FF map.

### 2.3. Fat Fraction Measurement

**Figure 1:**
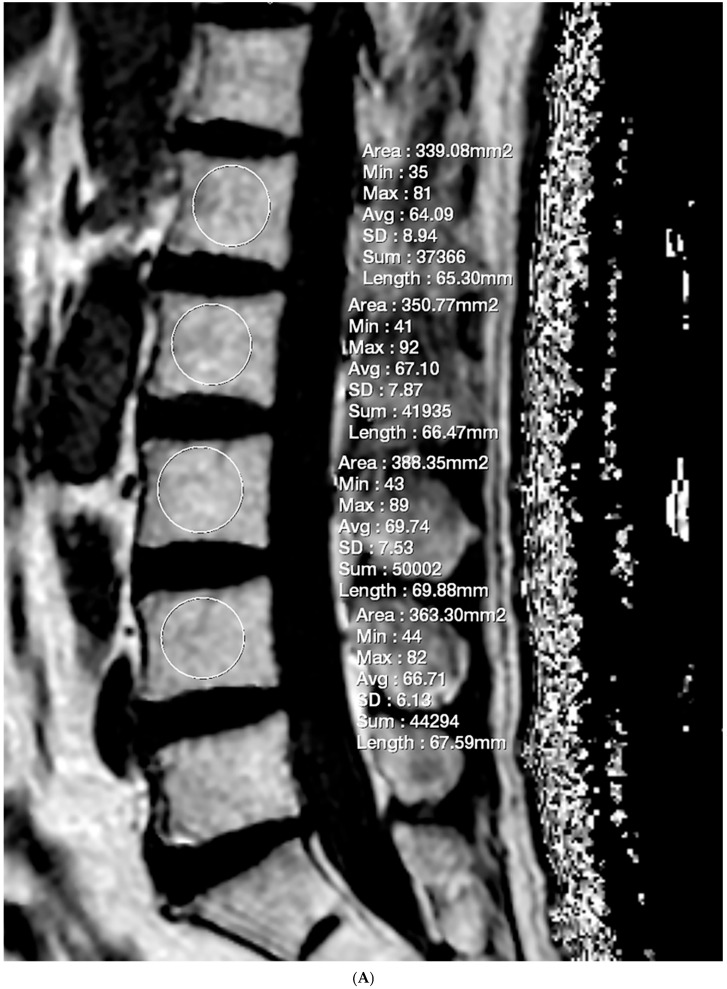

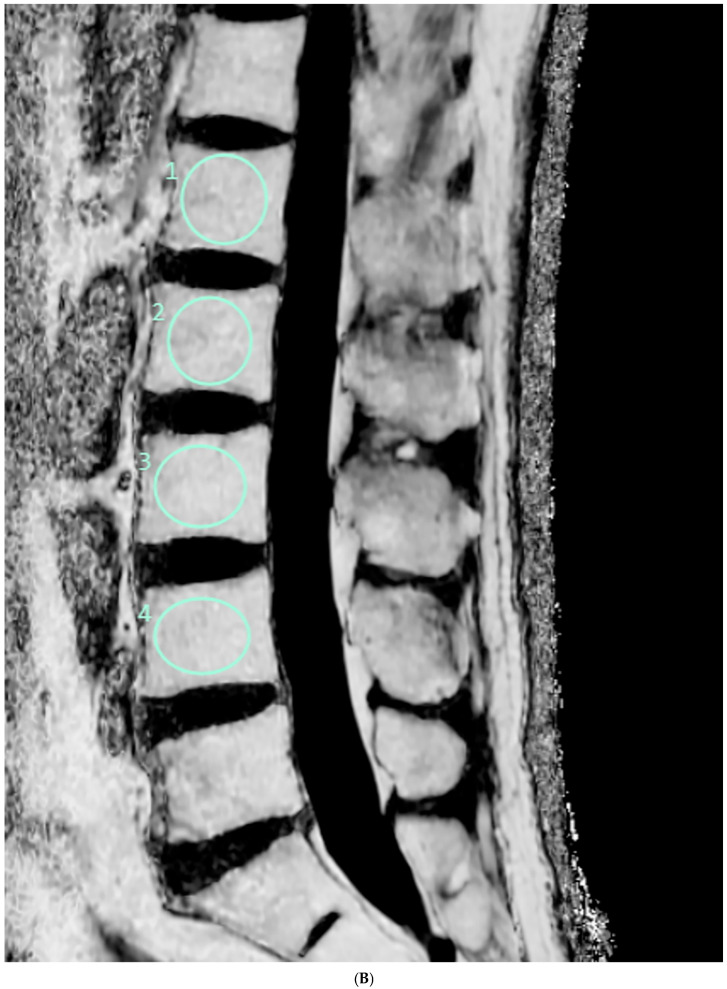

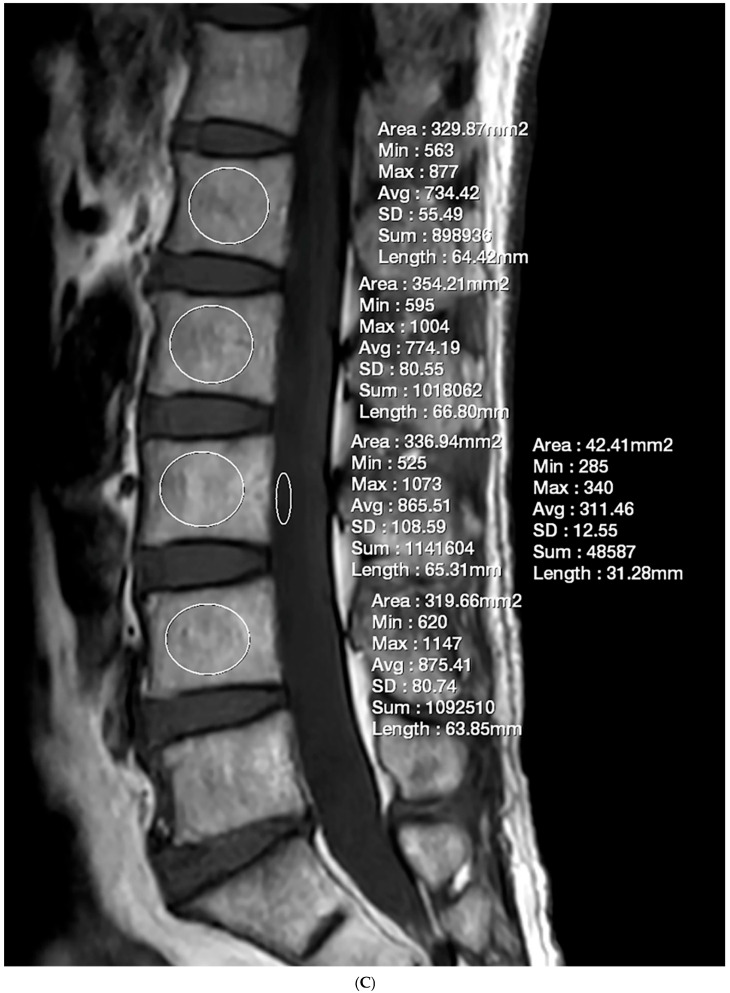

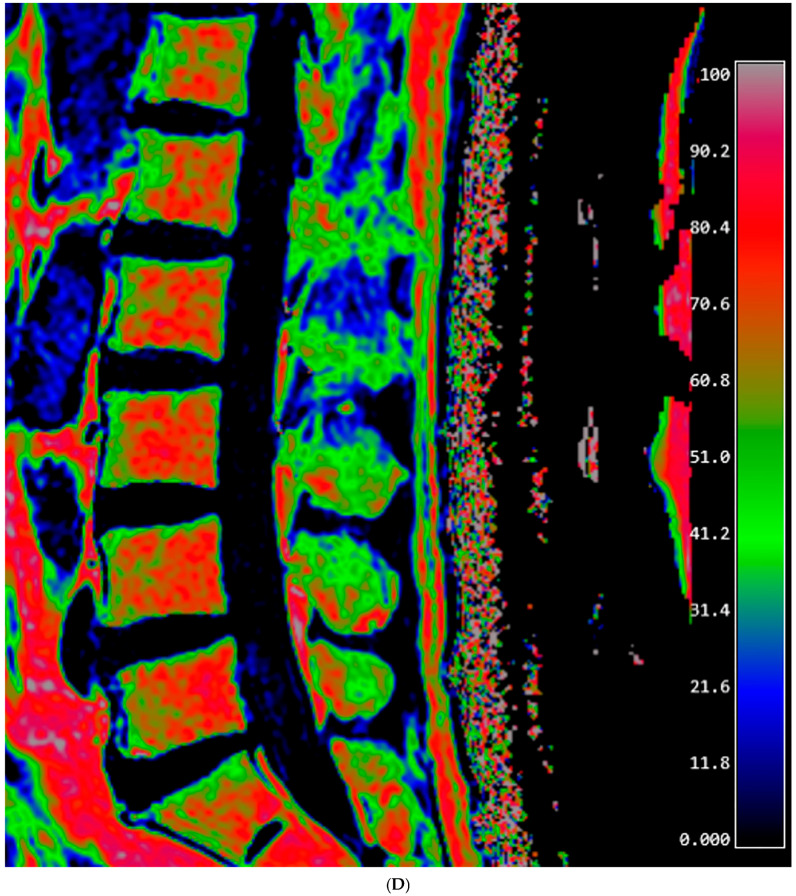

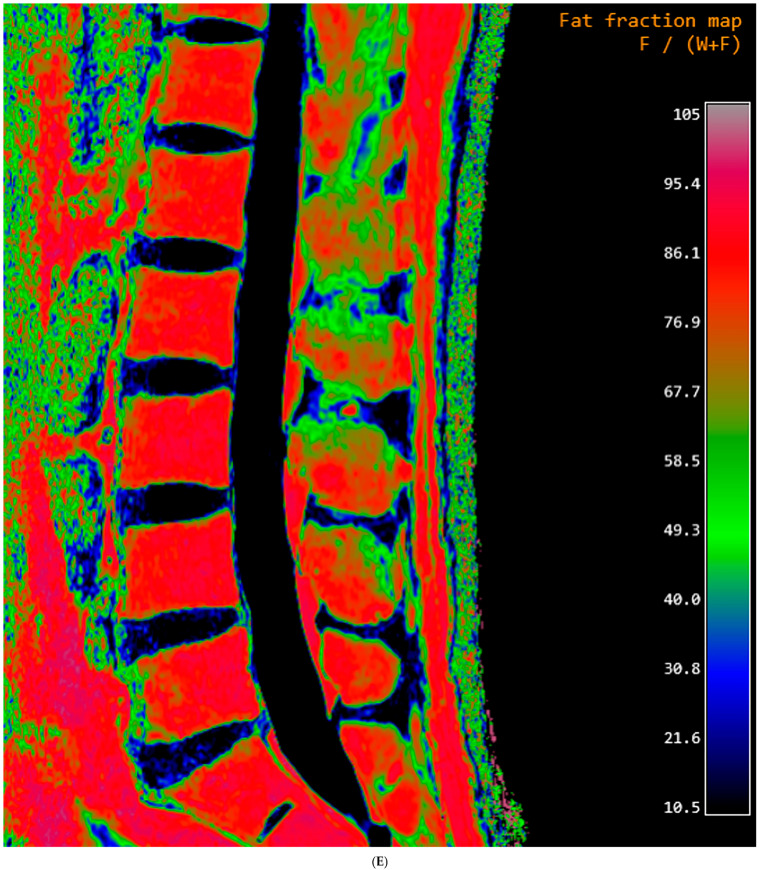
Lumbar spine MRI of a 59-year-old male. (**A**) Fat fraction (FF) measurement on gradient-echo (GRE)-based chemical-shift-encoded magnetic resonance imaging (CSE-MRI). The FF_GRE_ in this patient was 66.91%, representing the median value of the L1–L4 vertebral bodies. (**B**) FF measurement on spin-echo (SE)-based CSE-MRI. The FF_SE_ was 85.84%. The numbers in the figure indicate the ROI numbering in the EXPRESS program. (**C**) Measurement of the vertebral bone quality (VBQ) score was calculated by dividing the median value of T1-weighted signal of L1–L4 vertebral bodies by the cerebrospinal fluid (CSF) signal at the L3 level. The VBQ score for this patient was 2.63. (**D**,**E**) FF color maps were created through GRE- and SE-based CSE-MRI.

The FF measurement was independently performed by two radiologists who were unaware of the clinical information. Firstly, the FF measurements in GRE-based CSE-MRI were conducted using the INFINITT picture archiving and communication system. Elliptical regions of interest (ROIs) were drawn on the FF map, excluding the posterior venous complex and cortical bone, to encompass cancellous bone as much as possible (Figure 1). ROIs were delineated to the mid-sagittal image. In cases where obtaining information from the central image was challenging, ROIs were drawn on parasagittal images. Measurements were conducted on the L1–L4 bodies, and, in cases where abnormalities invaded all sagittal slices, this level was excluded from the measurements. The median values of the measurements were utilized as representative values (FF_GRE_). Next, the FF measurement in SE-based CSE-MRI was determined using diffusion analysis software (EXPRESS version 1.0, Philips Healthcare, Seoul, Republic of Korea). This program generates a fat fraction map by simply dividing the signal from the fat-only image by the sum of the signals from the water-only and fat-only images. After creating the FF map using mDixon-XD images, using the same criteria and methodology as in GRE-based CSE-MRI measurements, FF values were obtained from L1–L4 bodies (Figure 1), and the median was used as the representative value (FF_SE_).

### 2.4. Vertebral Bone Quality Score Measurement

The VBQ score measurement was performed on sagittal T1-weighted images using the method described by Ehresman et al. [16]. Similarly to FF measurements, elliptical ROIs were independently drawn by two investigators on the midline sagittal image (Figure 1). If measurements were challenging on the mid-sagittal image, they were either taken on the parasagittal image or excluded from the assessment following the same criteria as the FF measurement. The median signal intensity (SI) of L1–L4 bodies was chosen as the representative value (SI_VB_). Additionally, the SI of ventral cerebrospinal fluid (CSF) was measured at the L3 level (SI_CSF_). If severe stenosis prevented the measurement of CSF SI at the L3 level, measurements were taken at the L2 or L4 level. The VBQ score was calculated by dividing SI_L1-L4_ by SI_CSF_.

### 2.5. Evaluation for Vertebral Lesions

Based on morphologic MRI findings, focal vertebral lesions were defined. Focal vertebral lesions were included when two radiologists independently reviewed the images without clinical information and consistently identified them as true lesions. In case of disagreement between the two radiologists, a third reader independently reviewed the images to determine whether to include the lesion. If an individual had multiple lesions, the largest one was selected when considering it as the same diagnosis. If deemed as different diagnostic entities, each lesion was individually selected. In cases where there was a prior history of biopsy for each lesion, histopathologic confirmation was employed as the diagnostic reference standard. In the absence of available biopsy, diagnosis was based on the following criteria of imaging findings.

1. The typical imaging finding in all conventional MRI sequences.
2. In cases of an acute vertebral fracture, findings diagnosed with CT or plain radiography, with accompanying trauma history.
3. In cases other than fractures, the typical imaging appearance in MRI was taken at least 6 months before or after the evaluation.

**Figure 2:**
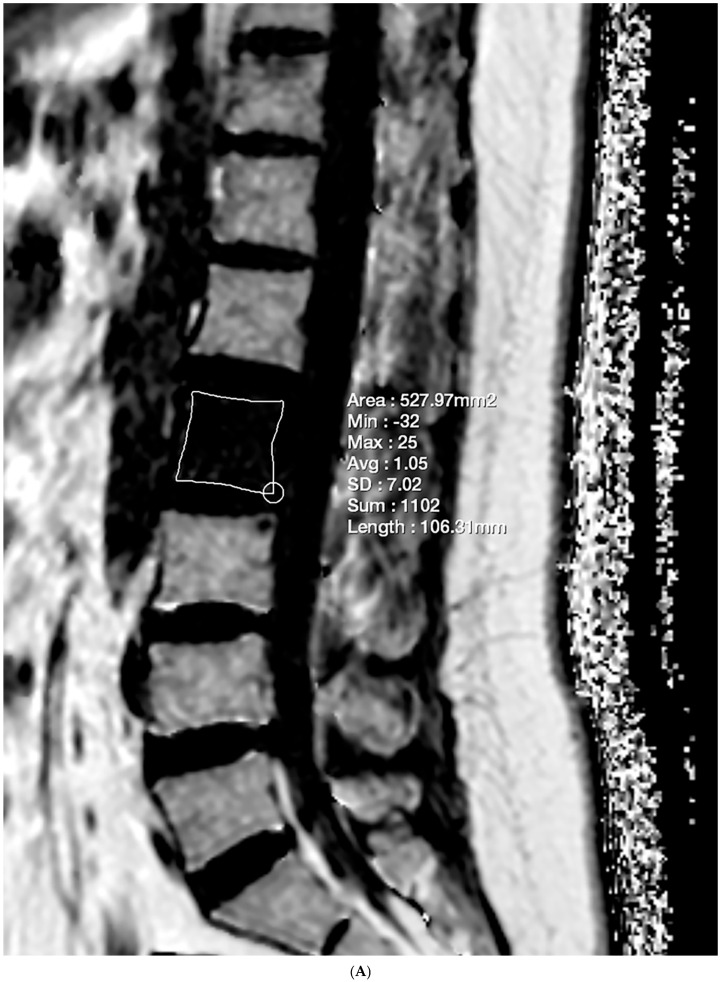

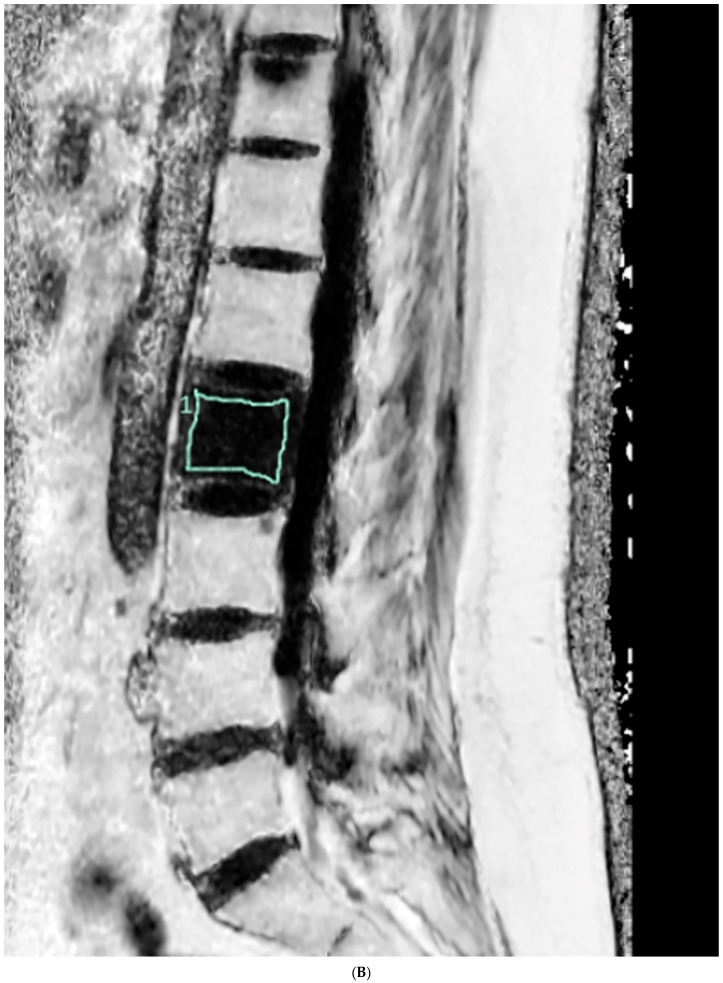

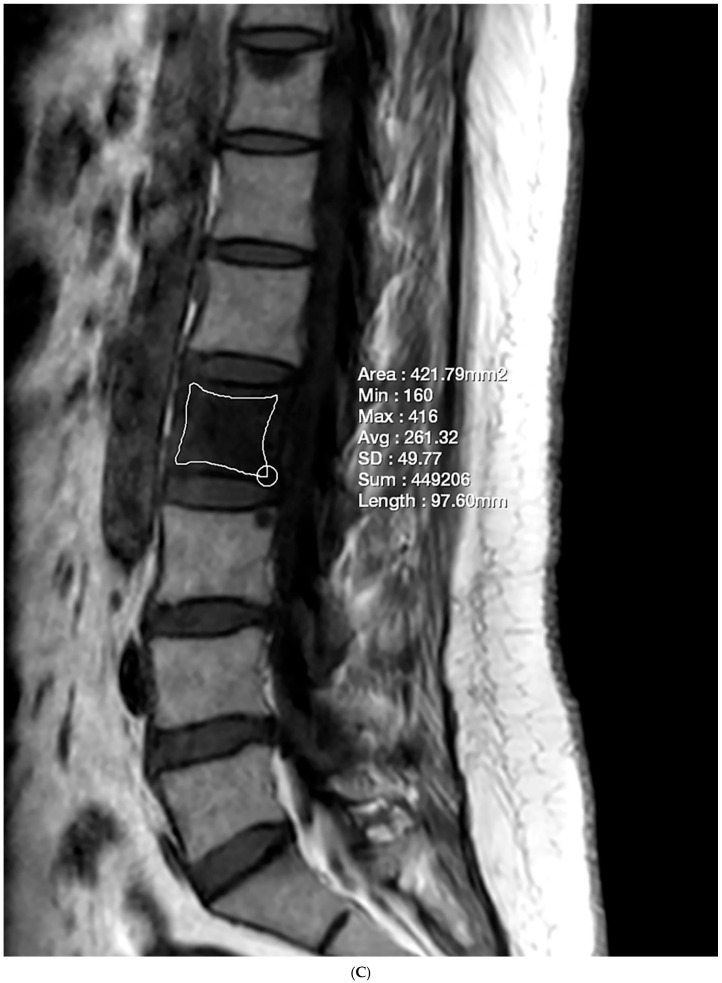
Lumbar spine MRI of a 48-year-old female with multiple bone metastases from adenoid cystic carcinoma of the parotid gland. (**A**) GRE-based CSE-MRI reveals an FF of 1.05% in the metastatic lesion of the L2 vertebral body. (**B**) On SE-based CSE-MRI, the FF of the corresponding lesion was 7.30%. The number in the figure indicate the ROI numbering in the EXPRESS program. (**C**) Multiple bone metastases are observed in the T11, L2, and L3 vertebral bodies on the T1-weighted image. The VBQ score for the L2 vertebral body lesion was 0.96.

Lesions meeting the criteria of the first and either the second or third condition were included in the evaluation. The imaging criterion standards were determined through a consensus reading by two researchers. Subsequently, the lesions were classified as either benign or malignant. Another researcher manually delineated the ROI on FF maps and sagittal T1-weighted images for each selected lesion to ensure maximum coverage, enabling the calculation of FF and VBQ scores (Figure 2).

### 2.6. Statistical Analysis

Statistical analyses were performed using commercially available software (MedCalc version 22.016; MedCalc Software, Ostend, Belgium). All continuous values were reported as the mean ± standard deviation. To assess reproducibility for each variable, an interobserver agreement was evaluated using the intraclass correlation coefficient (ICC) with a 95% confidence interval (CI). The correlation between FF_GRE_ and FF_SE_/VBQ score was analyzed using a linear regression and Pearson correlation (*r*). A Lin’s concordance correlation coefficient (ρ_c_) was used to measure the consistency between FF_GRE_ and FF_SE_. FF_GRE_ and FF_SE_ were compared using a paired *t*-test. To explore the correlation between age and each variable, Pa earson’s correlation was used. For benign and malignant vertebral lesions, the FF and the VBQ score were compared using the Mann–Whitney U test.

## 3. Results

The number of patients who met the inclusion criteria was 380. Out of these, 36 patients were excluded because of errors in GRE (*n* = 13) or SE (*n* = 2) CSE-MRI, hematopoietic disorder (*n* = 1), fracture (*n* = 12), infection (*n* = 1), tumor (*n* = 2), degeneration (*n* = 3), prior surgery (*n* = 1), or radiation treatment (*n* = 1). Therefore, a total of 344 patients were included, with 152 males and 192 females, and the average age was 59.7 ± 16.8 years. The mean FF and VBQ score of the study population are shown in Table 1.

### 3.1. Reproducibility

The ICCs were 0.979 (95% CI, 0.973–0.983) for FF_GRE_ and 0.992 (0.989–0.993) for FF_SE_, demonstrating excellent reliability for FF. The ICC for VBQ score was 0.949 (0.937–0.959), also indicating a high level of agreement. More specifically, the ICCs for SI_VB_ and SI_CSF_ were 0.993 (0.992–0.995) and 0.956 (0.946–0.965), respectively.

### 3.2. Correlation Between FF_GRE_ and FF_SE_/VBQ Score

**Figure 3:**
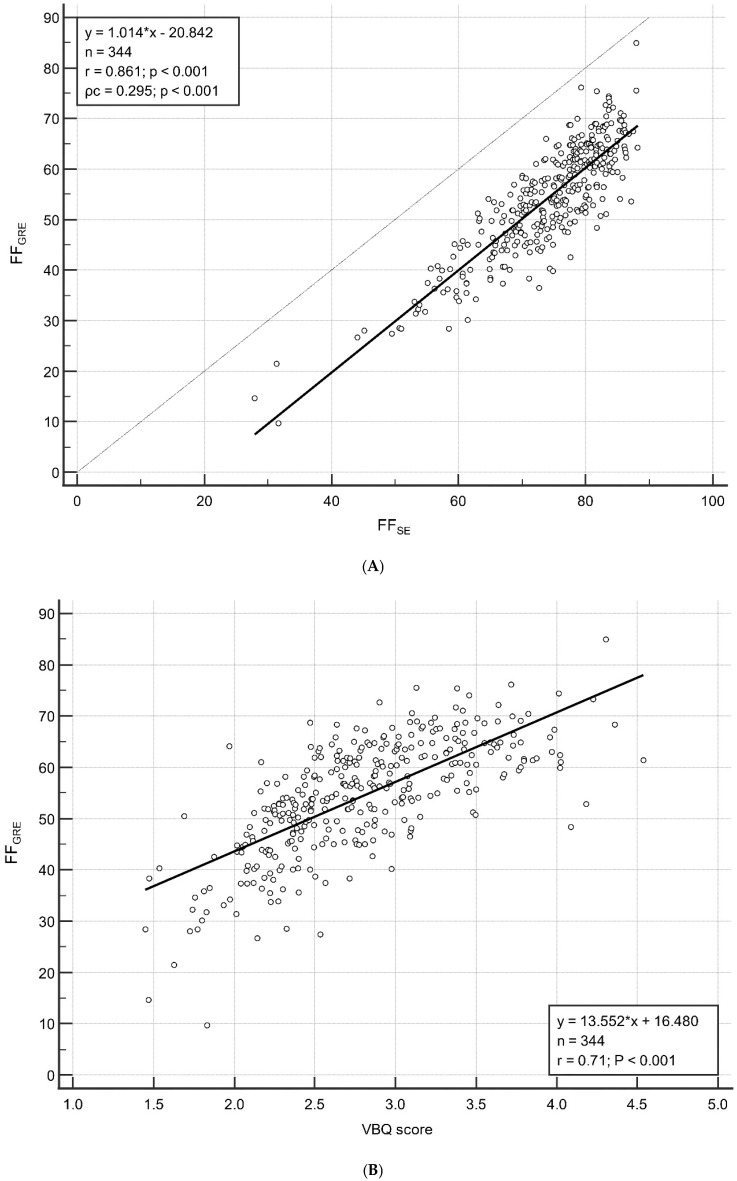
The correlation between FF_GRE_ and FF_SE_ (**A**), the VBQ score (**B**).

Figure 3 shows the correlation between FF_GRE_ and FF_SE_/VBQ score. According to linear regression analysis, FF_GRE_ exhibited strong correlations with FF_SE_ (*r* = 0.861; *p* < 0.001) and the VBQ score (*r* = 0.708; *p* < 0.001). Although the correlation between FF_GRE_ and FF_SE_ was high, the consistency between the two variables was not favorable. A Lin’s concordance correlation coefficient (ρ_c_) of 0.295 (*p* < 0.001) indicated a poor correlation between FF_GRE_ and FF_SE_ (Figure 3). When comparing the means, FF_SE_ was measured to be 19.82% higher than FF_GRE_ (54.31% vs. 74.13%; *p* < 0.001).

### 3.3. Correlation Between Age and FF/VBQ Score

Age and FF_GRE_ exhibited a moderate correlation (*r* = 0.583; *p* < 0.001). Both FF_SE_ and the VBQ score showed a somewhat lower yet still moderate correlation to FF_GRE_ (*r* = 0.477 and 0.468, respectively; both *p* < 0.001). When dividing participants by age (50 years at the threshold), FF_GRE_, FF_SE_ and the VBQ scores exhibited a lower correlation in the older group than the younger group (Table 2).

### 3.4. Vertebral Lesions Analyses

In total, 41 lesions were demonstrated in 40 patients. Hemangioma was the most prevalent with 15 cases, followed by acute vertebral fracture (*n* = 13), focal nodular marrow hyperplasia (*n* = 9), metastasis (*n* = 2), multiple myeloma (*n* = 1), and spondylodiscitis (*n* = 1). One patient had both hemangioma and focal nodular hyperplasia. Among these, multiple myeloma and spondylodiscitis were histopathologically confirmed. Two metastases originated from the adenoid cystic carcinoma of the parotid gland and adenocarcinoma of the lung, with multiple bone metastases in the spine. Biopsy confirmed bone metastasis in the left humerus and T8 body, respectively. Among the six types of lesions, metastases and multiple myeloma were classified as malignant, while the others were categorized as benign lesions.

**Figure 4:**
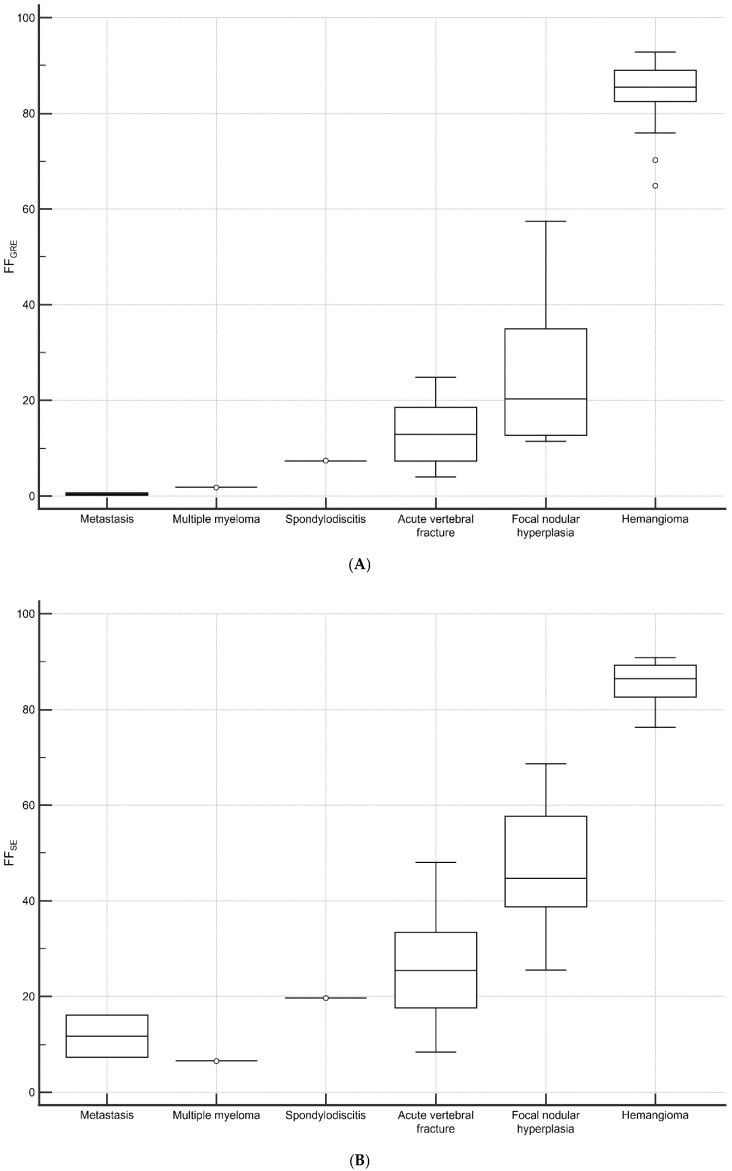

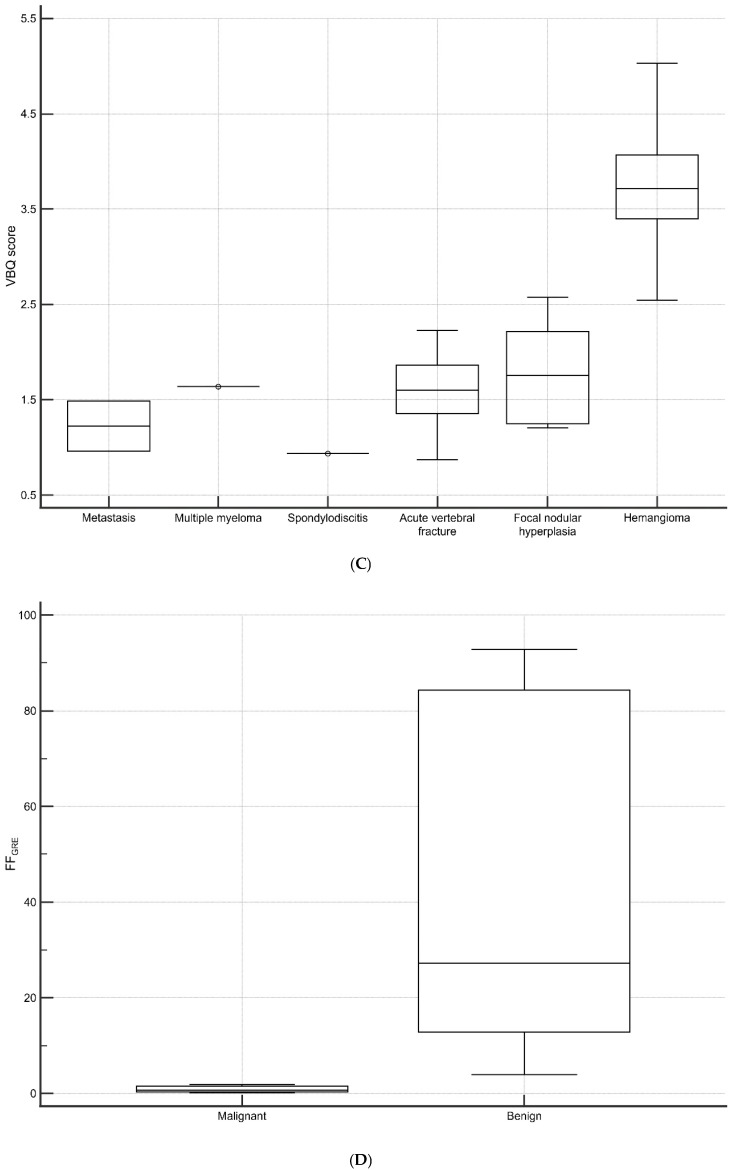

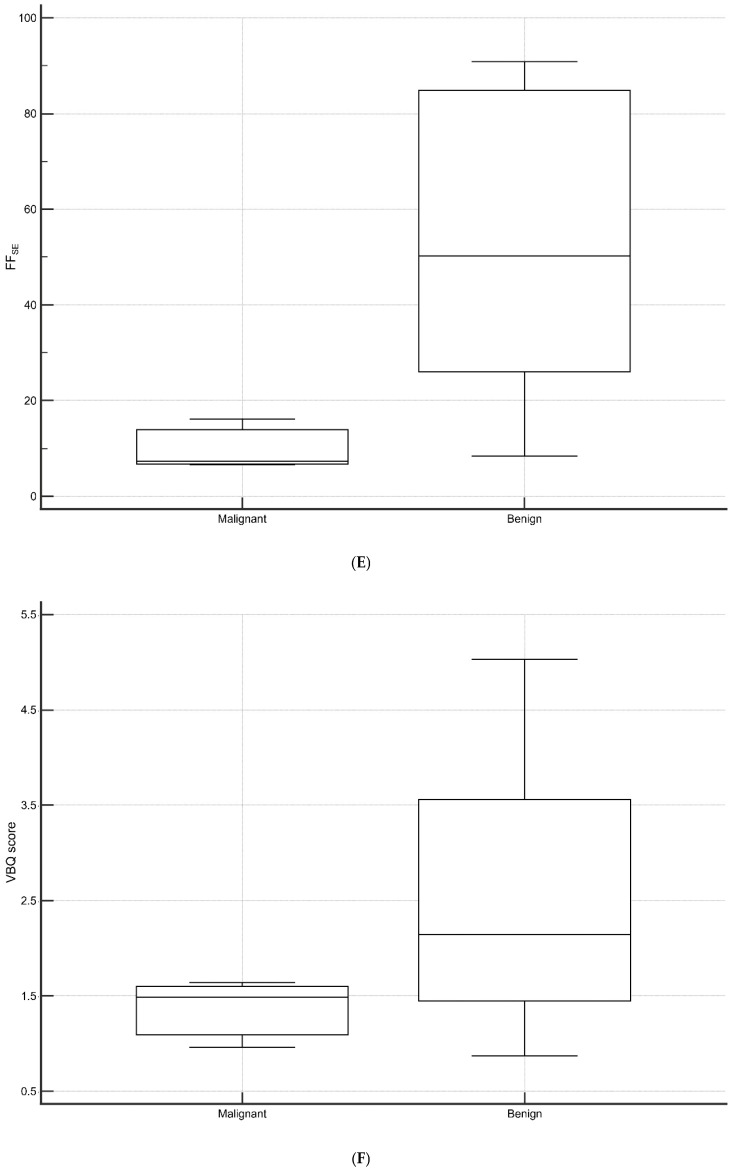
(**A**–**C**) The distribution of FF_GRE_ (**A**), FF_SE_ (**B**), and the VBQ score (**C**) in vertebral lesions. (**D**–**F**) The differences between malignant and benign lesions in FF_GRE_ (**D**), FF_SE_ (**E**), VBQ score (**F**). There was a statistically significant difference between FF_GRE_ and FF_SE_ (*p* = 0.004 and 0.007, respectively), but the VBQ score did not show a significant difference (*p* = 0.089).

FF and the VBQ score for each lesion were presented in Figure 4. Benign lesions exhibited an FF_GRE_ of 44.11 ± 34.29%, while malignant lesions showed 0.91 ± 0.86%. The two groups showed statistically significant differences (*p* = 0.004). Benign lesions were statistically significantly higher than malignant lesions in FF_SE_ (54.46 ± 28.36% vs. 9.99 ± 5.32%; *p* = 0.007). However, the difference in VBQ score was not statistically significant (2.48 ± 1.18 vs. 1.36 ± 0.36; *p* = 0.089).

## 4. Discussion

Our research demonstrated excellent inter-reader agreement for FF_GRE_, FF_SE_, and the VBQ scores. FF_GRE_ exhibited a strong correlation with FF_SE_ and the VBQ scores. However, Lin’s concordance correlation coefficient showed a low value, indicating that the consistency between FF_GRE_ and FF_SE_ was not favorable. There was a somewhat higher correlation between age and FF_GRE_, but all three measures—FF_GRE_, FF_SE_, and the VBQ scores—exhibited a moderate correlation with age. Clinically, this could be used to evaluate of changes in vertebral bone quality and marrow fat content. In the analysis of vertebral lesions, FF showed significant differences between both benign and malignant cases, whereas the VBQ scores did not show a statistically significant difference.

The CSE-MRI technique allows for the simultaneous acquisition of water and fat signals, enabling fat quantification [23]. However, there are multiple confounders such as T1 bias, T2* shortening effect, and multiple peaks in the fat spectrum that can interfere with accurate fat quantification [7,9,24]. The six-echo GRE CSE-MRI technique, which we adopted as the standard for fat fraction, has been reported to demonstrate high accuracy by correcting for these confounders [7,9]. On the contrary, the SE-based CSE-MRI technique did not correct for these confounders. As a result, the SE-based CSE-MRI technique leads to inaccurate fat quantification, contributing to a high correlation but low concordance between the two techniques. In other words, while FF_SE_ tends to show high values when FF_GRE_ is high (high correlation), the values of FF_GRE_ and FF_SE_ are not the same and exhibit differences (low concordance). As shown in Figure 3A, FF_SE_ is generally higher compared to FF_GRE_. These results are consistent with findings from a recent study [25]. The statistically significant difference, despite measuring the same FF, indicates that it is not feasible to simply replace FF_GRE_ with FF_SE_.

T1-weighted SE images are excellent for evaluating the cellular component of bone marrow. The SI of bone marrow is determined by the proportion of yellow marrow, which is composed of fat, and red marrow, which is composed of hematopoietic elements [26,27]. However, it is important to note that yellow marrow is not exclusively composed of fat, and red marrow also contains fat [3,28]. As the FF increases, there is a likelihood of an increase in SI on T1-weighted images, indicating a higher proportion of yellow marrow. However, precise measurement can be challenging. The correlation coefficient of 0.708 for the VBQ score in our study appears to reflect the influence of this background knowledge. Another weakness of the VBQ score is using of SI_CSF_ to correct SI_VB_. Despite strong interobserver agreement, the VBQ score concordance was relatively lower than FF, a trend noted in previous studies [11,16,17,18,29,30]. Our study found a lower concordance in SI_CSF_ compared to SI_VB_, aligning with prior research [18]. A small SI_CSF_ ROI may affect accuracy and stability. Unlike the fixed vertebral body, CSF flow may impact SI_CSF_ heterogeneity. The diverse degrees of stenosis in the CSF space and the varying conditions of the cauda equina among patients can also influence the measurements. Therefore, to enhance the interobserver agreement of VBQ scores, more detailed criteria for measuring SI_CSF_ may be needed.

There is a tendency for an increase in the volume of bone marrow fat with aging and osteoporosis [31,32,33]. In our study as well, age showed statistically significant positive correlations with both FF and the VBQ score. However, FF_GRE_ showed a slightly higher correlation with FF_SE_ and the VBQ score in our study. This difference is likely indicative of variations in the accuracy of fat quantification, as mentioned earlier. One important factor to consider is that, given that the elderly are primarily at higher risk for osteoporosis, the fact that the correlation of FF or the VBQ score decreases in this age group raises further questions as to whether these indicators can be reliably used in discriminating osteoporosis.

According to previous studies, FF has been reported to show a significant difference between malignant and benign lesions [7,13]. This is because malignant neoplasms completely replace cellular and fatty components, while benign lesions retain the fatty component [34,35]. We assumed that the VBQ score, which operates on a similar principle as FF, could also discriminate between benign and malignant lesions. The research results showed that there was a statistically significant difference in FF in both SE and GRE sequences, but the VBQ score did not exhibit such a difference. Our study suggests that, although the accuracy may be somewhat limited, not only FF_GRE_ but also FF_SE_ may potentially assist in distinguishing between benign and malignant lesions. However, considering the theoretical background that, in malignant lesions, the fatty component is replaced by cellular components, leading to a decreased in FF, relatively high and inaccurate FF values obtained from SE may cause misdiagnoses. Therefore, caution is required when interpreting these results. Further studies involving a larger patient cohort, including ROC analysis to determine the appropriate cut-off values, are necessary. There was no difference in the VBQ scores; however, this may be due to a limitation, given the small number of malignant lesions. Additional evaluation with a larger number of cases is necessary.

This study has several limitations. First, this study was conducted as a retrospective study, so there may be a selection bias. For example, there may be sex differences in FF inside the vertebral bodies, but gender was not considered in the selection of patients. Additionally, our study includes a very limited number of benign or malignant lesions. In particular, since the malignant lesions include only two metastases and one multiple myeloma, it is difficult to consider these as representative of malignant lesions. Additionally, although spondylodiscitis is a benign lesion, there is a report indicating that it may show low FF [36]. Therefore, future research should involve a larger number of benign and malignant cases and further investigate the differences in FF and the VBQ scores according to specific pathologic conditions. Second, while the VBQ score was developed as a tool to detect osteoporosis, our study did not consider its relationship with osteoporosis. The VBQ score and FF have been used for osteoporosis screening through MR [9,11,16]. However, to our knowledge, there has not been a study comparing these methods in the same patient cohort. It would be beneficial to evaluate osteoporosis screening using both the VBQ score and FF in the same patients. However, this falls outside the scope of our study and would require further research in the future. Third, many vertebral lesions were not histologically confirmed. However, especially for typical benign lesions, it is ethically challenging to confirm them histologically, and this limitation must be accepted as inherent.

## 5. Conclusions

In conclusion, FF_GRE_ showed a high correlation with both FF_SE_ and the VBQ score. Nevertheless, replacing FF_GRE_ with FF_SE_ proves to be challenging. All three indicators showed a moderate positive correlation with age. Unlike FF, the VBQ score could not distinguish between benign and malignant lesions.

## Figures and Tables

**Table 1:** Mean FF and VBQ scores of the study population.

	Mean ± Standard Deviation	*p*
FFGRE	54.31 ± 11.04	<0.001
FFSE	74.13 ± 9.37	
VBQ score	2.79 ± 0.58	

**Table 2:** The correlation between age and FF/VBQ score.

	FF_GRE_		FF_SE_		VBQ Score	
	*r*	*p*	*r*	*p*	*r*	*p*
All (*n* = 344)	0.583	<0.001	0.477	<0.001	0.468	<0.001
Age < 50 (*n* = 79)	0.538	<0.001	0.488	<0.001	0.372	0.001
Age ≥ 50 (*n* = 265)	0.199	0.001	0.111	0.070	0.183	0.003

FF_GRE_, fat fraction (FF) measured using gradient-echo based chemical shift-encoded MRI (CSE-MRI); FF_SE_, FF measured using spin-echo based CSE-MRI; the VBQ score, vertebral bone quality score.

## Data Availability

The data presented in this study are available on request from the corresponding author. The data are not publicly available due to the need for approval from the affiliated institution’s DRB (Data Review Board), which is required for disclosure or export.

## Abbreviations

BMAT	Bone marrow adipose tissue
CSE-MRI	Chemical shift-encoded magnetic resonance imaging
GRE	Gradient-echo
FF	Fat fraction
SE	Spin-echo
VBQ	Vertebral bone quality
TR	Repetition time
TE	Echo time
FOV	Field of view
ROIs	regions of interest
SI	Signal intensity
CSF	Cerebrospinal fluid
CI	confidence interval
